# Case Report: The ultrasound features of acquired cystic disease-associated renal cell carcinoma: a case series

**DOI:** 10.3389/fonc.2023.1187495

**Published:** 2023-05-26

**Authors:** Yanrong Yang, Hongyan Chen, Yongzhong Li, Jiaojiao Zhou

**Affiliations:** Department of Ultrasound, West China Hospital of Sichuan University, Chengdu, China

**Keywords:** ACD-RCC, image, ultrasound, pathology, case

## Abstract

**Background:**

Acquired cystic disease-associated renal cell carcinoma (ACD-RCC) is a new subtype listed by the 2016 World Health Organization (WHO) classification, which occurred in end-stage renal disease (ESRD) patients. This study will present the imaging characteristics of the four cases diagnosed with ACD-RCC. Ultrasound is expected to help detect abnormalities early in the follow-up of patients on regular dialysis, allowing patients to receive early treatment.

**Case presentation:**

We searched the pathology database of our hospital for all inpatients diagnosed with ACD-RCC between January 2016 and May 2022. Pathology, ultrasound, and radiology readings are performed by experienced physicians with the title of attending physician or higher. Four cases were included in this study, all of whom were male, aged from 17 to 59. Two cases suffered from ACD-RCC in both kidneys, and kidney nephrectomies were performed. One case underwent renal transplantation, whose creatinine was back to normal, and the rest were on hemodialysis. On the pathological images, heteromorphic cells and oxalate crystals can be seen. Both ultrasound and enhanced CT showed an enhancement of the solid component of the occupancy. We followed up with outpatient and telephone visits.

**Conclusion:**

In clinical work, ACD-RCC should be considered when the mass appears in the background of multiple cysts in the kidney in patients with ESRD. A timely diagnosis will help with treatment and prognosis.

## Introduction

Acquired cystic disease-associated renal cell carcinoma (ACD-RCC) is a new subtype in the light of the 2016 World Health Organization (WHO) classification, which occurred in end-stage renal disease (ESRD) patients (1). Dialysis is one of the measures to aid or replace the renal function in ESRD patients, which may result in the development of acquired cystic kidney disease (ACKD), defined as more than three cysts per kidney, or cysts accounting for >25% of the renal parenchyma in a patient without polycystic kidney disease (2). Compared with the general population, patients with ACKD have a more than 100-fold increased risk of developing RCC (3). The main diagnosis form of ACD-RCC is pathological biopsy and immunohistochemistry. In the 2016 guideline, a microcystic, sieve-like, and unique cribriform architecture and an eosinophilic and/or clear cytoplasm and prominent nucleoli can be seen in ACD-RCC (1). Studies have found that the oxalate crystals, expression of AMACR, and positive for the CD10 marker were the characteristics of ACD-RCC (1, 2, 4), while in other subtypes such as clear cell renal cell carcinoma (ccRCC), it is characterized by tumors with reticulated septa composed of narrow thin-walled blood vessels, with clear cytoplasm and variable sized nuclei, and expressed PAX8 markers in the nucleus (5). However, the biopsy core diagnostic rate was 80% (6), which limited its clinic diffusion. Radiomics, whose current applications include the differentiation of benign from cancerous kidney tumors and another differentiation diagnosis, has improved early diagnosis compared with traditional radiology diagnosis. Radiomics such as machine learning texture analysis or ensemble deep learning model combined with CT as well as molecular imaging performed better than radiologists’ assessment (6). Studies of the ultrasound signature of ACD-RCC are rare. In this study, we will present four cases of ACD-RCC, based not only on pathological biopsy and immunohistochemistry features but also on image features, with the aim of providing a reference for clinic work.

## Case presentation

All procedures performed in studies involving human participants were in accordance with the ethical standards of the institutional and/or national research committee(s) and with the Helsinki Declaration (as revised in 2013). The need for informed consent was waived.

All inpatients diagnosed with ACD-RCC from January 2016 to May 2022 were searched in the pathology database of our hospital. Experienced physicians with the title of attending physician or higher performed ultrasound, radiology, and pathology imaging reading. A total of four cases (four male patients aged from 17 to 59) were included, all of whom had a history of CKD. Two cases received nephrectomies due to bilateral ACD-RCC, while the remaining two underwent unilateral nephrectomies. One of the cases underwent a kidney transplant and his creatine levels returned to normal, while the rest were on hemodialysis.

### Case 1

**Figure 1:**
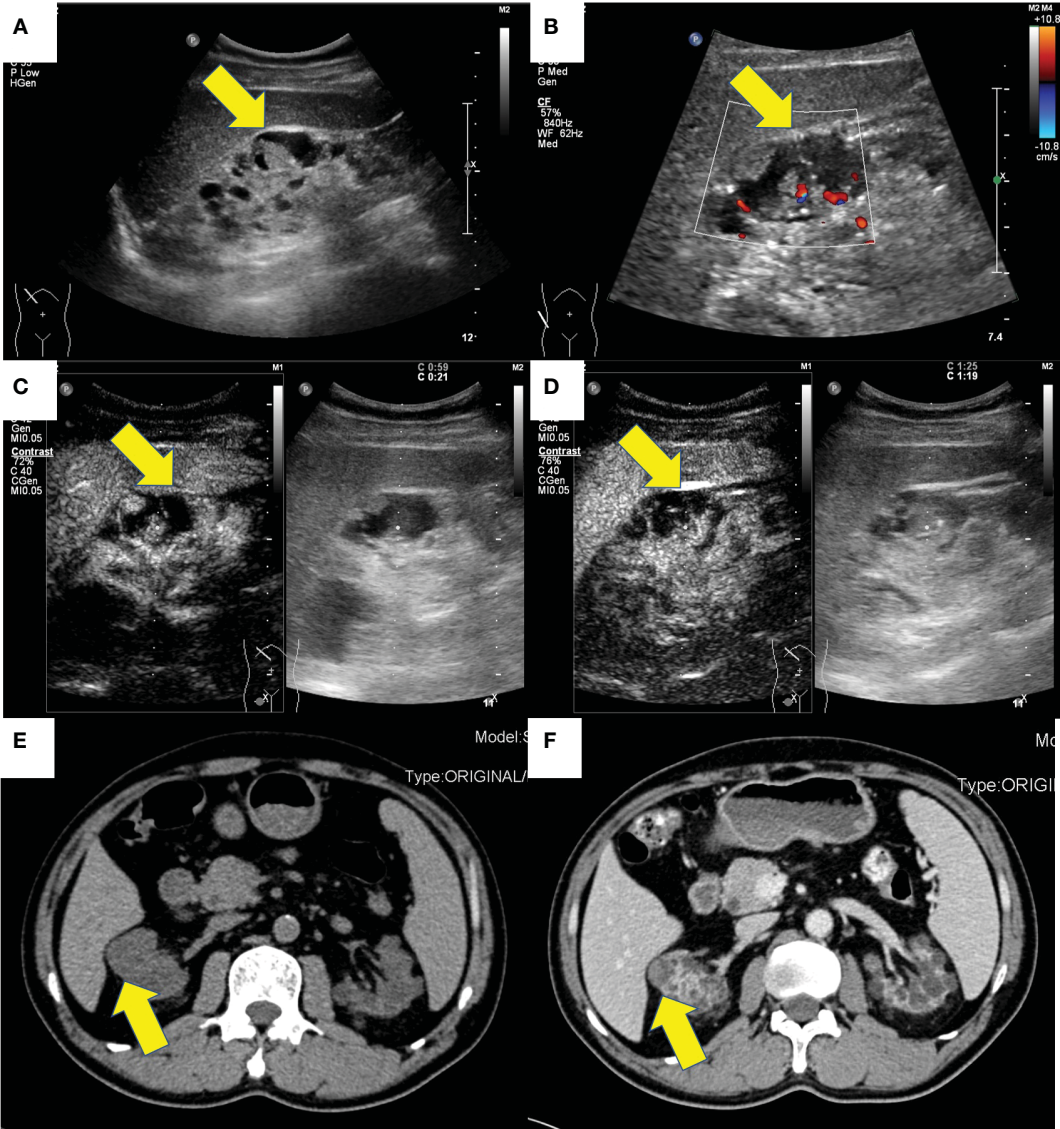
The ultrasound and CT images of a 58-year-old male (case 1). **(A)** Gray-scale, **(B)** color Doppler, **(C)** cortical phase and **(D)** medullary phase sonograms of the mass in right kidney. **(E)** non-enhanced CT and **(F)** contrast-enhanced CT of the mass of right kidney.

**Figure 2:**
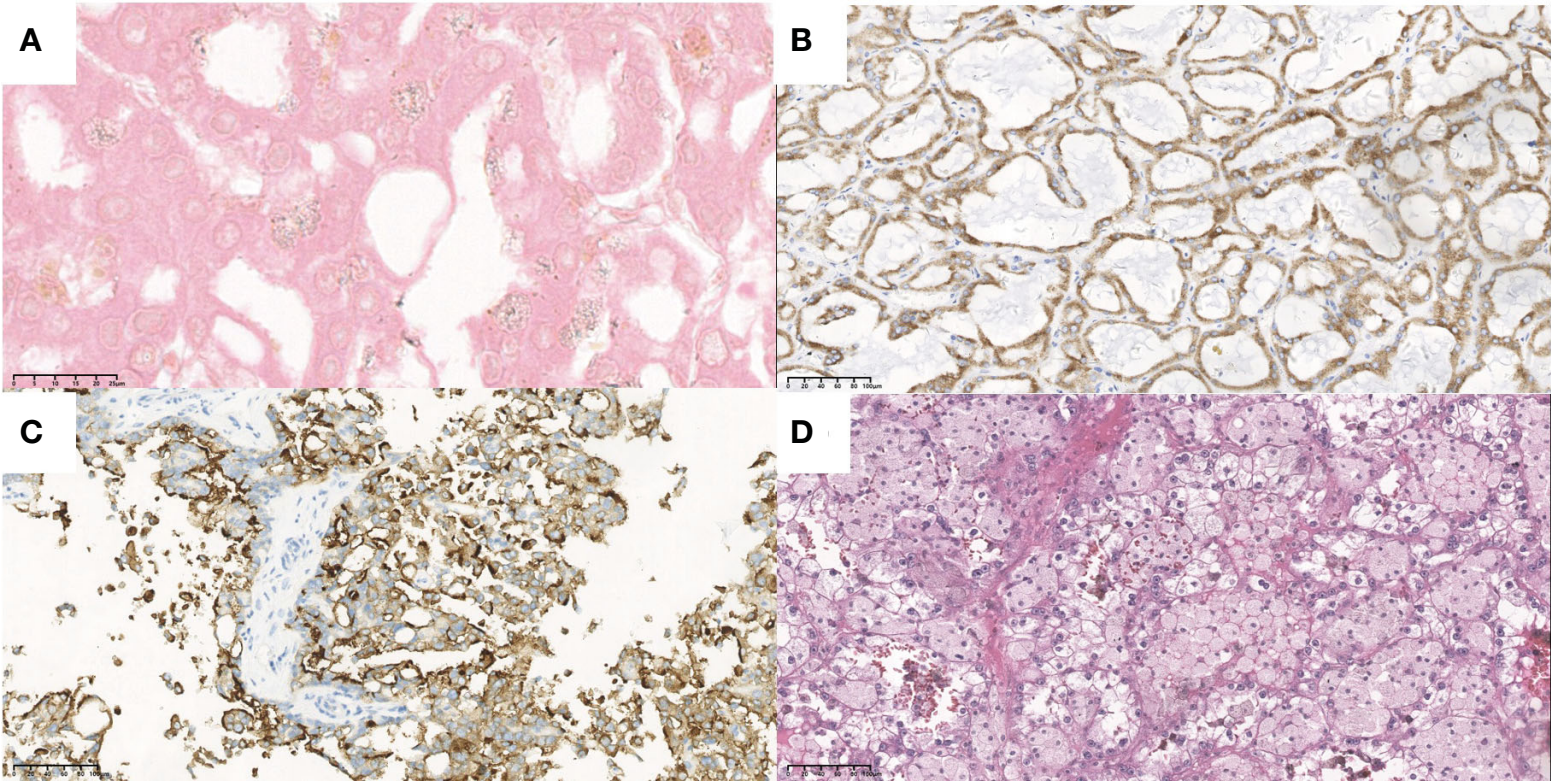
The typical pathological and immunohistochemistry figures of these four cases. **(A)** the oxalate crystalline deposits (x100) and **(B)** the positive AMACR marker (x20) of the tumor in case 1. **(C)** the positive CD10 marker (x20) of the tumor in case 3. **(D)** the eosinophilic and prominent nucleoli (x20) of the tumor in case 4.

Case 1 is a 58-year-old man diagnosed with chronic kidney disease (CKD) 20 years ago, in regular hemodialysis for more than 15 years, and renal hypertension for more than 20 years, who was admitted to the hospital in 2017 for a right renal occupancy. The contrast-enhanced ultrasonography (CEUS) of the kidney (sulfur hexafluoride microspheres) demonstrated that the parenchyma of both kidneys was echogenically enhanced, with indistinct corticomedullary demarcation. A cystic-solid mixed echogenic nodule of approximately 1.8 × 1.5 cm in size with clear borders and regular morphology was detected in the middle part of the right kidney, which was deeply enhanced in the cortical phase and low enhanced in the medulla phase. Multiple cysts were detected in both kidneys, with well-defined borders and regular morphology, and dotted echogenicity was seen in part of the capsule wall. Acquired cystic lesions and possible tumor lesions in the right kidney were considered in the enhanced CT. The ultrasound and enhanced CT images are shown in **Figure 1**. A laparoscopic radical right nephrectomy was performed. The oxalate crystals under light microscopy and the expression of immunohistochemical markers such as AMACR, CD10, SDHB, FH, CAIX, EMA, and CK7 suggest a preference for ACD-RCC. In 2019, he was readmitted for occupation of the left kidney and an abdominal CT scan revealed the possibility of acquired cystic kidney disease in the left kidney. Thinking of his history of ACD-RCC in his right kidney, a laparoscopic radical nephrectomy of the left kidney was performed, and a pathological biopsy suggested that it was ACD-RCC, where there was renal cell carcinoma (CA) with adenoid cells, eosinophilic cytoplasm, some vacuoles in the cytoplasm, and multiple foci of oxalate crystalline deposits in the surrounding renal cells (**Figure 2A**). Immunohistochemistry demonstrated that the marker of SDHB, PAX8, FH, CD10, and AMACR was expressed (**Figure 2B**). Now, he receives hemodialysis three to four times a week.

### Case 2

**Figure 3:**
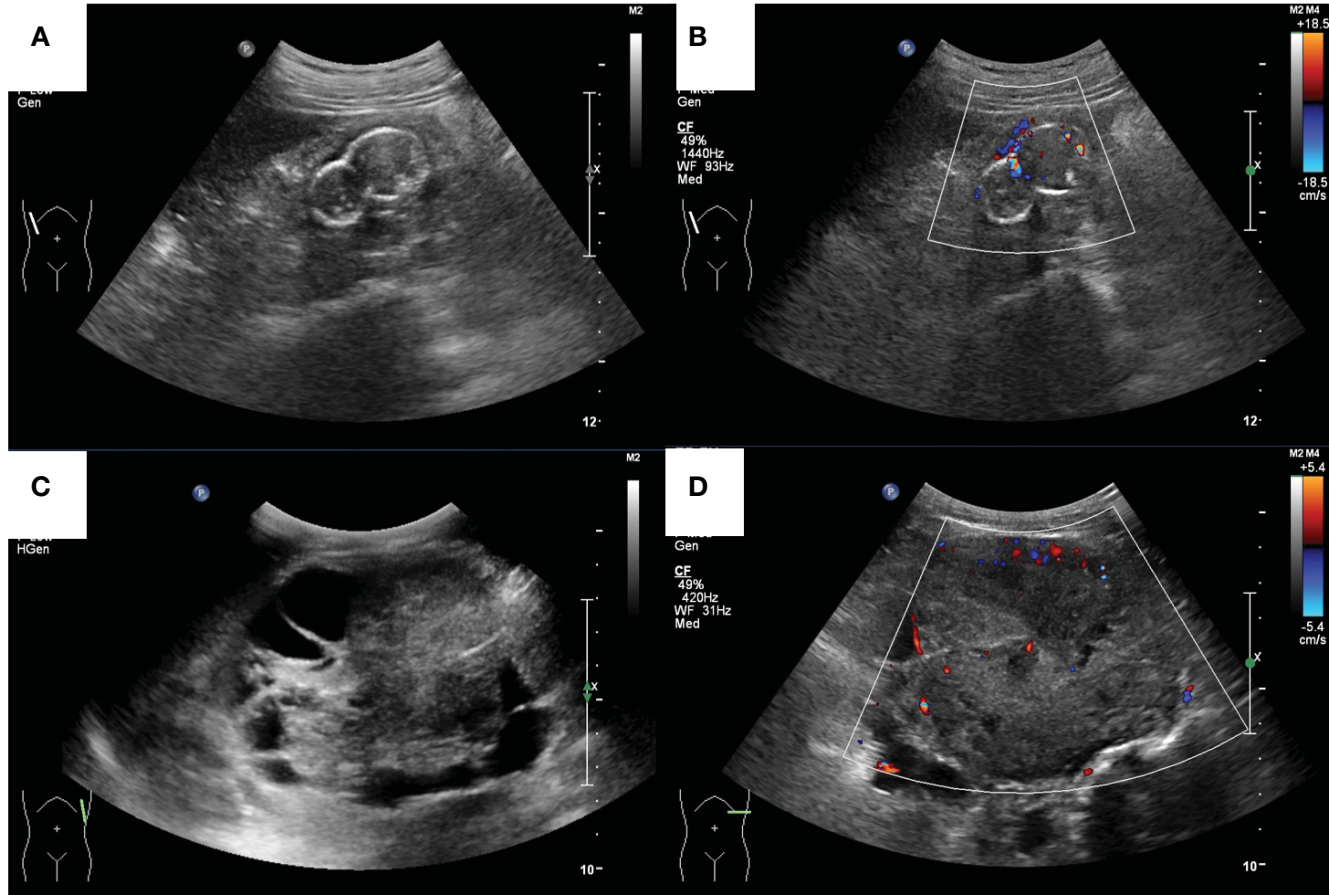
The ultrasound images of a 39-year-old male (case 2) and a 17-year-old male (case 4). **(A)** Grey-scale and **(B)** color Doppler sonograms of the mass in the right kidney in case 2. **(C)** Grey-scale and **(D)** color Doppler sonograms of the mass in the left kidney in case 4.

Case 2, a 39-year-old man with a history of CKD for more than 10 years and a history of hemodialysis for more than 7 years, was admitted to the hospital in 2016 for a physical exam that revealed an occupied right kidney. CT examination suggested that “a lobulated mass protruded from the lower lateral part of the right kidney with calcified spots inside and partially enhanced under the background of chronic nephritis, where the kidney cancer cannot be excluded.” The ultrasound examination showed a hypoechoic mass sized 4.1 × 2.5 × 3.1 cm, where there was sparse blood flow, surrounded by ring-like echogenic with dot echogenic focus inside under the background of multiple cysts (**Figures 3A, B**). Laparoscopic radical resection was performed on the right kidney tumor. Histological and immunohistochemical images, with expressed markers EMA, PAX2, PAX8, CD9, Ksp-cadherin, and SDHB, suggested that it accorded to ACD-RCC. He is currently on dialysis every Monday, Thursday, and Saturday.

### Case 3

Case 3 was a 56-year-old man, who had a history of maintenance hemodialysis for CKD. He received a left nephrectomy for the ruptured left kidney with hemorrhage in the local hospital in 2017 (before the nephrectomy, he was on hemodialysis for 7 years). Post-operative pathology and immunohistochemistry showed that it accorded to ACD-RCC. He presented to the local hospital again in 2018 for right side back pain. CT demonstrated multiple cysts in the right kidney and the possibility of right kidney hemorrhage. He then underwent a nephrectomy on his right kidney. Post-operative pathology and immunohistochemistry of the right kidney demonstrated that there was calcium oxalate crystal deposition, and expressed CK7, RCC, CD10, PAX2, and CAIX immunohistochemical markers, which accorded with ACD-RCC (**Figure 2C**). Until now, he has been on twice-weekly hemodialysis.

### Case 4

Case 4 was a 17-year-old man with a history of CKD for more than 10 years and regular peritoneal dialysis for more than 8 years. In 2017, abdominal ultrasound suggested a heterogeneous hypoechoic mass sized 8.8 × 7.2 × 6.7 cm in the left kidney, with a visible border, irregular shape, and a few blood flow signals inside, which was suspected as a tumorigenic lesion (**Figures 3C, D**). Enhanced CT suggested a mass in the lower pole of the left kidney with heterogeneous enhancement, considered to be tumorigenic. The pathological finding that nests of anomalous cells were detected (**Figure 2D**) and expressed immunohistochemistry markers EMA, CAIX, CK8/CK18, FH, SDHB, and PAX-8 suggested that ACD-RCC was considered. In 2018, laparoscopic radical left nephrectomy was performed, and in 2020, renal transplantation was performed. His recent biochemistry demonstrated that creatinine was 68 μmol/L, and eGFR was 134.41 ml/min/1.73 m^2^.

The total information of these four cases is listed in **Table 1**.

**Table 1:** The total imaging information of these four cases.

	Clinic information							Imaging information					
Case number	Sex	Age	Pre-operation Scr (μmol/L)	Pre-operation eGFR (ml/min/1.73 m^2^)	Duration of preoperative dialysis (years)	Current status	Pathogenesis of ESRD	Size (cm)	Morphology	Border	Vascular	The enhancement mode of CEUS	The enhancement mode of CT
Case 1	M	58	1,177	3.69	15 (hemodialysis)	Regular hemodialysis	Primary glomerulonephritis	1.8 × 1.5	Regular	Clear	Hypovascular	High enhanced in cortical period and low enhanced in medulla period	No enhancement
Case 2	M	39	1,224	3.99	12 (hemodialysis)	Regular hemodialysis	Primary glomerulonephritis	4.1 × 2.5 × 3.1	Irregular	Clear	Hypovascular	NA	Partially enhanced
Case 3	M	56	899	6.16	7 (hemodialysis)	Regular hemodialysis	Primary glomerulonephritis	NA	NA	NA	Hypovascular	NA	No enhancement
Case 4	M	17	637	10.48	8 (peritoneal dialysis)	Kidney transplant status	Nephrotic syndrome	8.8 × 7.2 × 6.7	Irregular	Visible	Hypovascular	NA	Heterogeneously enhanced

Scr, serum creatinine; eGFR, estimated glomerular filtration rate; NA, not available; ESRD, end-stage renal disease.

## Discussion

ACD-RCC was a new subtype listed by WHO in 2016, whose incidence in the pathology literature was 32% and 38% (7). The main pathologic features, the gold diagnosis criteria, are abundant eosinophilic cytoplasm, prominent nucleoli, and sieve-like architecture, with architectural heterogeneity as well as the common presence of calcium oxalate crystals (1). Some studies thought that calcium oxalate crystal depositions are a relatively common feature of end-stage kidneys, which are neither necessary nor sufficient for the diagnosis (2). Although unexpressed CK7 is typical (1), frequent expression of AMACR with focal expression of CK7, positivity for CD10, and RCC marker can also be seen in many cases (2, 4).

There were rare studies focused on the imaging characteristic of ACD-RCC. On imaging, ACD-RCCs are typically soft tissue-appearing masses (63%), with a number having cystic components (7). Berkenblit et al. reported that a single and unilateral lesion with a well-defined outline that protruded to the renal peritoneum was typically shown in ACD-RCC (8). Carnahan et al. reported that there was no specific pathognomonic finding for ACD-RCC, which demonstrated equivocal or mild enhancement on CT, which is consistent with our patients’ features, and T2 hypo-intensity and restricted diffusion in MRI (7). In the case report of Edo et al., the lesions, typically hypovascular masses, were commonly presented as an intra-cystic growth and/or as a solid mass that surrounds cysts, which demonstrated poor to slight enhancement on enhanced CT (9). In ultrasound imaging, the mass of ACD-RCC can have variable shapes (regular or irregular) and borders (clear or invisible), and their echoes were heterogeneous, in one of which there was calcification. However, in the four cases of our study, all these masses were hypovascular in color Doppler mode. The solid content of ACD-RCC, a type of malignant mass, would be enhanced in CT or CEUS, which can differentiate from other begin diseases. The detailed information about the ultrasound and CT imaging features is shown in **Table 1**. As we all know, ultrasound is an inexpensive and convenient examination. Meanwhile, the ultrasonic microbubble contrast agent is non-nephrotoxic, which is more suitable for ESRD patients compared with the enhanced CT, whose iodine-containing contrast agents may aggravate kidney damage. We suggest that patients who undergo regular hemodialysis receive routine ultrasonography every half a year or every year. Once they have clinical symptoms, such as back pain or hematuria, ultrasound examination is recommended. Ishikawa et al. reported that contrast-enhanced ultrasonography with perflubutane microbubbles can demonstrate blood flow in a tumor with no or poor enhancement on dynamic CT (10). In our study, we use sulfur hexafluoride microspheres as the contrast agent. The solid ingredients were enhanced to varying degrees during all the phases, which should be differentiated to clear cell renal cell carcinoma (fast-forward and fast/slow-retrograde) and papillary renal cell carcinoma (slow-forward and fast/slow-retrograde) (11). In clinical practice, radiologists should be vigilant when abnormally enhanced lesions were found under the background of multiple cysts.

Generally, ACD-RCC is considered to be indolent and has a favorable prognosis, whose standard treatment is partial or total nephrectomy (4). The NCCN panel recommends surgical excision by partial nephrectomy or radical nephrectomy for the management of clinical stage I-III renal masses, and for patients with relapsed or stage IV RCC, the therapy should be considered comprehensively according to the patients’ physical conditions (12, 13). A multi-institutional central pathology study that included 112 ACD-RCC patients found that the cancer-specific survival (CSS) and recurrence-free survival (RFS) rates of patients with ACD-RCC were comparable with those with ccRCC (ACD-RCC group: 5-year CSS: 93.7%, 5-year RFS: 95.6%; ccRCC group: 5-year CSS: 95.8%, 5-year RFS: 93.7%) and significantly better than those with the papillary subtype (5-year CSS: 77.9%, 5-year RFS: 85.4%) (14). Moreover, their results showed that a long dialysis duration and lymphovascular invasion were independent factors of the prognosis of ACD-RCC. They found that the favorable post-operative survival of ACD-RCC patients may be attributed to the fact that there was a higher proportion of individuals with stage 1 than other subtypes (14). Meanwhile, the regional lymphadenopathy at the surgery of ACD-RCC was 5.3%, and the distant metastases at the surgery of ACD-RCC was 2.6%, which were higher than clear cell carcinoma (1.8% and 1.8%, respectively) and lower than the papillary subtype (6.5% and 4.9%, respectively) (14). According to their data, the follow-up period was 77.3 ± 61.5 (1–255) months, and the cancer death rate was 6.2% (14). Therefore, early diagnosis is of great importance. Regular imaging screening for masses or large cysts is recommended for patients with ESRD. ACD-RCC can be seen as a kind of complex renal cystic lesion. To assess the malignant risk of complex renal cystic lesions, the Bosniak classification has been used, which is mainly based on contrast-enhanced CT imaging (CECT). In 2020, the European Federation of Societies for Ultrasound in Medicine and Biology (EFSUMB) updated a proposal for a contrast-enhanced ultrasound (CEUS)-adapted Bosniak cyst categorization. The 2019 CECT Bosniak classification and the 2020 EFSUMB CEUS Bosniak classification were different in some categories (15, 16). For ultrasound doctors, combining the two kinds of classification may make a more accurate diagnosis. Meanwhile, ACD-RCC should be differentiated from other cystic diseases, such as peripelvic cysts and parapelvic cysts. Peripelvic cysts are small, multiple, and confluent, and have an irregular shape with multiple and thin linear septa that extend radially from the renal hilum in ultrasound imaging (17). The parapelvic cysts, which are localized in the sinus, are single and larger compared to the peripelvic cysts.

There were some limitations in our study. Firstly, the number of cases is small. In the future, we will enlarge the sample size to acquire more generalizable ultrasound features to provide some reference for the early diagnosis of ACD-RCC. Secondly, some imaging examinations were absent, and only one case did CEUS. More cases are needed to summarize its imaging features.

## Conclusion

ACD-RCC occurs frequently in ESRD patients with ACD who generally have a lengthy history of dialysis. For these patients, a regular ultrasound or CT examination of the kidney system is necessary. In spite of its indolent behavior, a timely diagnosis will result in a favorable prognosis for the patient.

## Data availability statement

The raw data supporting the conclusions of this article will be made available by the authors, without undue reservation.

## Ethics statement

The studies involving human participants were reviewed and approved by Biomedical Ethics Review Meeting of West China Hospital of Sichuan University. Written informed consent was obtained from the patients/participants for the publication of this case report.

## Author contributions

YY performed the case series and wrote the manuscript. Besides, HC is the Equal & First author in this manuscript. HC and YL supported the data collection and manuscript revision. JZ supervised the writing and revision of the manuscript. All authors contributed to the article and approved the submitted version.
